# Protective Effect of CXCR7 Against Hypoxia/Reoxygenation Injury in Renal Tubular Epithelial Cells

**DOI:** 10.1007/s12013-024-01312-x

**Published:** 2024-05-28

**Authors:** Ping Meng, Chunli Liu, Jingchun Li, Ping Fang, Liling Chen

**Affiliations:** 1 Department of Central Laboratory, Huadu District People’s Hospital of Guangzhou, Guangzhou, Guangdong 510800 China; 2 Department of Clinical Laboratory, Huadu District People’s Hospital of Guangzhou, Guangzhou, Guangdong 510800 China; 3 Department of Clinical Laboratory, Sanya Hospital of Traditional Chinese Medicine, Sanya, Hainan 572000 China

**Keywords:** CXCR7, Renal tubular epithelial cells, H/R, Autophagy, Apoptosis

## Abstract

Acute kidney injury (AKI) is a multifactorial syndrome with complex pathophysiology and prognosis. Ischaemia‒reperfusion injury (IRI) is a major cause of induced AKI. The aim of this study was to investigate the effect of upregulated CXCR7 expression on renal tubular epithelial cell apoptosis induced by hypoxia/reoxygenation (H/R). HK-2 cells were divided into three groups: control group (pcDNA3.1), hypoxia/reoxygenation + pcDNA3.1 group (H/R+pcDNA3.1) and CXCR7 overexpression + hypoxia/reoxygenation group (H/R+ Flag-CXCR7). Protein levels of renal tubular epithelial cell injury-, apoptosis- and autophagy-related markers were assessed by qRT‒PCR, Western blotting, flow cytometry (FCM), immunofluorescence and transmission electron microscopy (TEM). In addition, HK-2 cells were treated with the autophagy inhibitor 3-MA and divided into 3 groups: control group, 3-MA + pcDNA3.1 group, and 3-MA + Flag-CXCR7 group. Changes in autophagy and apoptosis in renal tubule epithelial cells were assessed by Western blotting and FCM. Compared with those in the control group, the protein and mRNA expression levels of CXCR7 in HK-2 cells were significantly lower under H/R conditions. Under H/R conditions, CXCR7 overexpression in HK-2 cells significantly downregulated the expression of NGAL. Moreover, CXCR7 overexpression significantly decreased H/R-induced cleaved PARP-1 and cleaved Caspase 3 levels, increased the level of the antiapoptotic protein BCL-2 and the autophagy-related molecules ATG5 and LC3B II, and significantly inhibited the expression of P62. Autophagy flow and TEM also showed that CXCR7 significantly promoted autophagy. CXCR7 significantly alleviated the 3-MA-induced inhibition of autophagy and increase in apoptosis. Upregulated CXCR7 expression can inhibit renal tubular epithelial cell apoptosis and damage by regulating autophagy. In conclusion, CXCR7 is a promising target for the prevention and/or treatment of AKI.

## Introduction

Acute kidney injury (AKI) is a clinical syndrome of acute renal dysfunction caused by various aetiological factors and can be caused by renal ischaemia‒reperfusion (I/R), nephrotoxic drugs and sepsis. AKI manifests as a sharp decline in renal function, a decreased glomerular filtration rate, the accumulation of metabolites, environmental disorders and secondary systemic multiorgan system damage [1]. AKI is usually characterized by a rapid decrease in the glomerular filtration rate and an increase in the serum concentrations of urea nitrogen, creatinine, and proteinuria [2]. AKI has a high morbidity and mortality rate, with a global average of 23%, affecting approximately 20% of hospitalized patients and up to 60% of patients in intensive care units [3–5]. Despite recent insights into the causes and underlying mechanisms, there is still a lack of effective drugs for the treatment of AKI. Improving the prognosis of AKI patients is an important global health problem.

IRI is a major cause of AKI and a global health concern associated with high morbidity and mortality [6, 7]. To date, no specific interventions limit injury or improve recovery and survival. In addition, after I/R injury, tightly controlled autophagy is important for maintaining the viability of renal tubules after initial damage, and this process contributes to normal kidney repair and regeneration. Renal tubular epithelial cell injury is one of the main pathological features of AKI. Recent evidence demonstrates that neutrophil gelatinase- associated lipocalin (NGAL) is closely associated with AKI. Several experimental and clinical studies have shown that the expression of urine and serum NGAL increases significantly in AKI. In particular, the urine NGAL level is closely associated with the severity of kidney injury, and could be detected earlier than other AKI markers. However, the mechanism of renal tubular epithelial cell injury has not yet been elucidated and needs to be further explored.

Research on renal tubular epithelial cell injury in AKI has focused mainly on regulative cell death, autophagy changes, and cell cycle arrest [8]. In addition, AKI induces cell death pathway activation through apoptotic and necrotic mechanisms in tubular epithelial cells [9]. Autophagy is a normal homeostatic cellular process that removes dysfunctional components and damaged organelles and recycles cellular proteins through the lysosomal degradation pathway [10, 11]. Autophagy, which is associated with several diseases and must be tightly controlled to maintain cellular homeostasis, is known to occur in response to either intracellular or extracellular factors, such as extremely stressful starvation conditions, hypoxia, endoplasmic reticulum stress or oxidative stress, organelle damage, and pathogen infections [12]. It has been reported that a deficiency or excess of autophagy-related proteins contributes to several diseases. Compared with WT mice, the selective deletion of Atg5 or Atg7 in the proximal tubules of mice resulted in progressive kidney damage and increased tubular cell apoptosis and tubulointerstitial fibrosis [13, 14]. SQSTM1/p62 is a scaffold protein closely involved in the macroautophagy process. p62 functions to anchor the ubiquitinated proteins to the autophagosome membrane, promoting degradation of unwanted molecules [15]. Despite these findings, the regulatory mechanisms of autophagy and apoptosis in AKI are poorly understood.

CXC chemokine receptor family (CXCR family) proteins are members of the G protein-coupled receptor (GPCR) family and include CXCR1 ~ 7, which play important roles in various physiological and pathological processes, such as the inflammatory response, angiogenesis, and tumour metastasis [16]. CXCR7, a recently discovered CXCL12 receptor, has a greater affinity for CXCL12 than does the classical receptor CXCR4. With further research, CXCR7 has been shown to play an important role in a variety of diseases and tumours [17–19]. In recent years, an increasing number of studies have demonstrated that CXCR7, CXCR4 and their ligand CXCL12 play important roles in kidney disease [20]. Our previous study showed that CXCR7 plays a crucial role in inhibiting renal fibrosis [21]. Another study showed that CXCR7 alleviates cardiac insufficiency after myocardial infarction by promoting angiogenesis and reducing apoptosis [22]. CXCR7 silencing has been shown to reduce the proliferative ability and enhance the apoptosis rate of ESCs [23]. The administration of SDF-1β to diabetic rats induced by a high-fat diet followed by a small dose of streptozotocin has been shown to significantly reduce cardiac apoptosis [24]. CXCR7 protects against brain cerebral I/R injury [25]. The relationship between CXCR7 and autophagy in AKI has not been reported.

In this study, our group investigated the role of CXCR7 in tubular epithelial cells (TECs) under hypoxic stress. We established a hypoxia/reoxygenation (H/R) model of human HK-2 tubular epithelial cells to investigate the effects and mechanisms of CXCR7 on HK-2 cell injury and apoptosis. Elucidating the involvement of CXCR7 in hypoxic stress might provide new mechanistic insights for a potential biomarker and diagnostic and therapeutic target of AKI.

## Methods

### Materials

Human HK-2 renal tubular epithelial cells were purchased from Wuhan Punosei. DMEM/F-12 (1:1) medium and 0.25% trypsin-EDTA (1X) were purchased from Gibco Australia Origin. FBS and ECL luminescent Solution were obtained from Invigentech, and DAPI staining solution and 4% PFA were obtained from Beyotime. The transfection reagent EX FECT Transfer, RNA extraction kit, and qRT‒PCR mix were purchased from Vazyme. The protein extraction and quantitation reagents were obtained from Abcam. The anti-GAPDH, anti-CXCR7, anti-NGAL, anti-cleaved PARP-1, anti-P62, anti-ATG5, anti-LC3B, anti-cleaved caspase3, anti-BCL-2, and anti-Flag antibodies were obtained from Abmart. 3-MA was obtained from MedChemExpress.

### Cell Culture

Human HK-2 cells were cultured in DMEM/F-12 (1:1) supplemented with 10% PBS in a thermostatic incubator at 37 °C in 5% CO_2_ and 95% O_2_. The cell hypoxia/reoxygenation (H/R) models were divided into three groups: normal control group (pcDNA3.1), H/R group (pcDNA3.1 + H/R) and CXCR7 overexpression group (pFlag-CXCR7 + H/R).

To overexpress CXCR7, HK-2 cells at 60–70% confluence were transfected using transfection reagents according to the manufacturer’s protocol. The culture medium was changed 24 h posttransfection before H/R.

The 3-MA models were divided into three groups: normal control group (pcDNA3.1), 3-MA group (pcDNA3.1 + 3-MA) and CXCR7-overexpressing group (pFlag-CXCR7 + 3-MA). Cells were treated with 5 mM 3-MA for 24 h after transfection.

### H/R Model

The medium of the HK-2 cells was replaced with serum-free medium, and the cells were placed in an anoxic device for anoxic culture in 5% CO_2_, 1% O_2_ and 94% N_2_ for 12 h and then cultured normally for 6 h.

### qRT‒PCR

After treatment, total RNA was extracted from cells by using an RNA extraction kit according to the manufacturer’s instructions, and the RNA was reverse transcribed into cDNA by using a reverse transcription kit according to the manufacturer’s instructions. cDNA was subsequently used as a template for qRT‒PCR. The following primers were synthesized by BGI: CXCR7 forward primer 5′-AGCACAGCCAGGAAGGCGAG-3′ and reverse primer 5′-TCATAGCCTGTGGTGGTTGGC-3′; and GAPDH forward primer 5′-GCACCGTCAAGGCTGAGAGGAAC-3′ and reverse primer 5′-TGGTGAAGAGCGCCAGTGGA-3′.

### Flow Cytometry (FCM)

Apoptotic cells in all groups were detected by flow cytometry. After treatment, the cells were cultured for 48 h, collected, digested with trypsin, and washed with PBS, after which 5 μl of Annexin V-FITC was added in accordance with the instructions of the Annexin V-FITC/PI apoptosis kit. After incubation at 37 °C for 30 min in the dark, 10 μl of PI was added, and the percentage of apoptotic cells was immediately assessed by flow cytometry.

### Western Blot Analysis

HK-2 cells were lysed with RIPA buffer to extract cellular protein, and the protein concentration was determined using a BCA protein quantification assay. Protein samples was subjected to polyacrylamide gel electrophoresis, transferred to a synthetic membrane, and incubated with primary/secondary antibodies. The grey values of the protein bands were visualized using ECL luminescence and imaged and analysed with ImageJ software.

### Laser Confocal Microscopy

The cells were washed with precooled PBS buffer twice to remove the residual culture medium, and the cells were then fixed in 4% paraformaldehyde (PFA) at room temperature for 15 min. Then, the cells were washed with PBS three times to remove the excess fixing solution. The cells were incubated with DAPI for 10 min to stain the nuclei, followed by two washes with PBS. The cells were sealed with sealing tablets. Images were taken under a laser confocal microscope.

### Transmission Electron Microscopy (TEM)

Cells were collected by centrifugation, fixed at room temperature for at least 2 h with 2.5% glutaraldehyde, and placed in a refrigerator at 4 °C. Afterwards, the cell samples were processed and imaged by TEM.

### Statistical Analysis

GraphPad Prism 9.5 statistical software was used for the analyses. The t test was used for analyses between two groups, and one-way analysis of variance was used for comparisons among multiple groups. *P* < 0.05 indicated statistical significance.

## Results

### CXCR7 Expression was Downregulated in H/R-induced HK-2 Cells

**Fig. 1:**
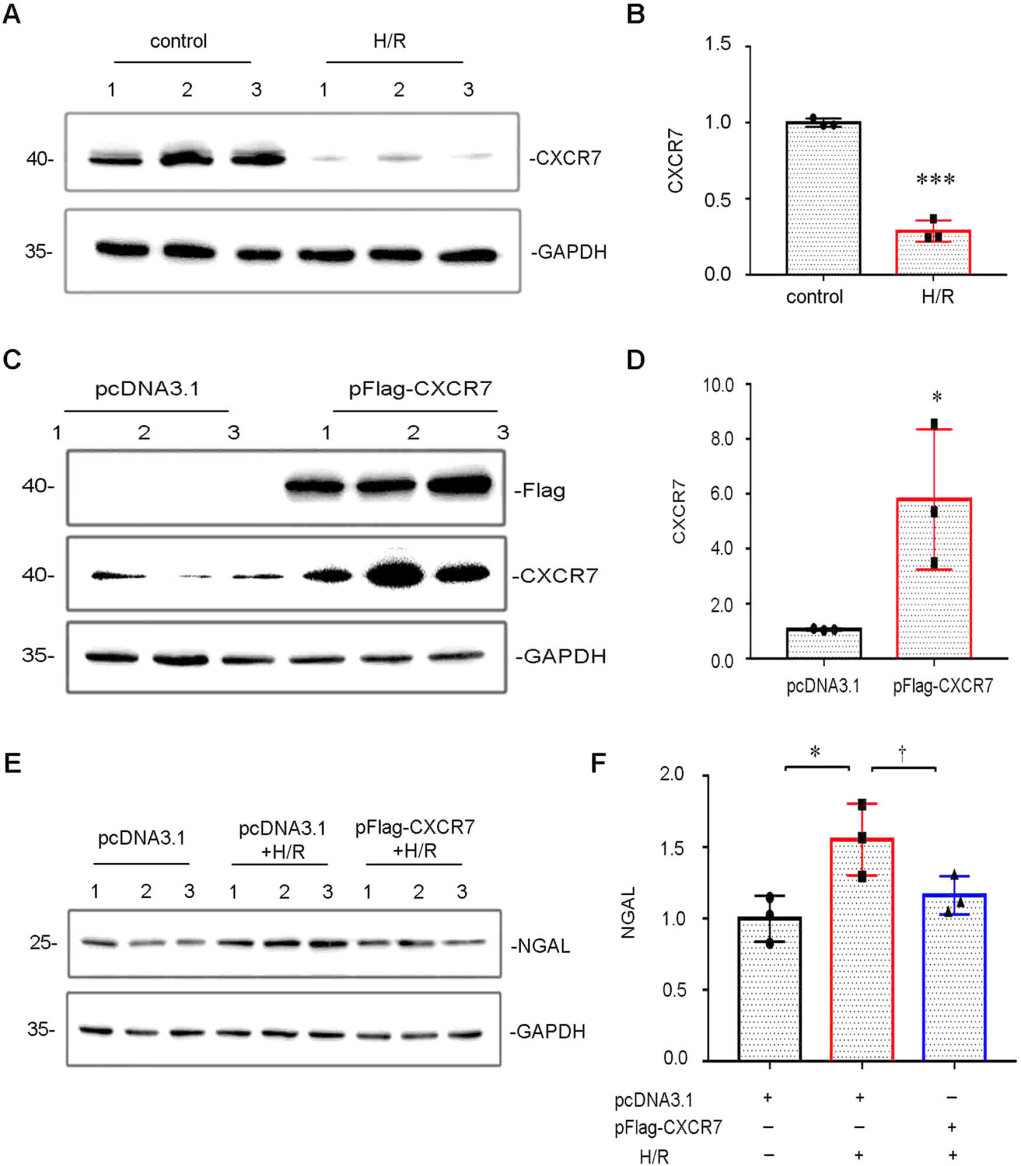
CXCR7 is downregulated in HK-2 cells under HR conditions. **A**, **B** Representative Western blots showing CXCR7 protein levels in HK-2 cells under HR conditions. ***P < 0.001, *n* = 3. **C**, **D** Protein level of CXCR7 in pcDNA3.1- and pFlag-CXCR7-overexpressing HK-2 cells, as determined by Western blotting. *P < 0.05, *n* = 3. **E**, **F** The protein levels of NGAL in HR+pFlag-CXCR7-treated HK-2 cells. *P < 0.05, †p < 0.05, *n* = 3

The Western blot and qRT‒PCR results showed that, compared with that in the control group, the protein expression of CXCR7 in HK-2 cells under H/R conditions was significantly lower (Fig. 1A, B). HK-2 cells were transfected with the CXCR7 overexpression plasmid pFlag-CXCR7, and Western blot results showed that the overexpression was successful, with a significant difference between the non-transfected and transfected cells (Fig. 1C, D). Western blot analysis revealed that after CXCR7 was overexpressed, the protein expression of NGAL, a marker of renal tubular injury, significantly decreased, indicating that the upregulation of CXCR7 expression alleviated H/R-induced renal tubular epithelial cell injury (Fig. 1E, F).

### CXCR7 Overexpression Mitigates Apoptosis

**Fig. 2:**
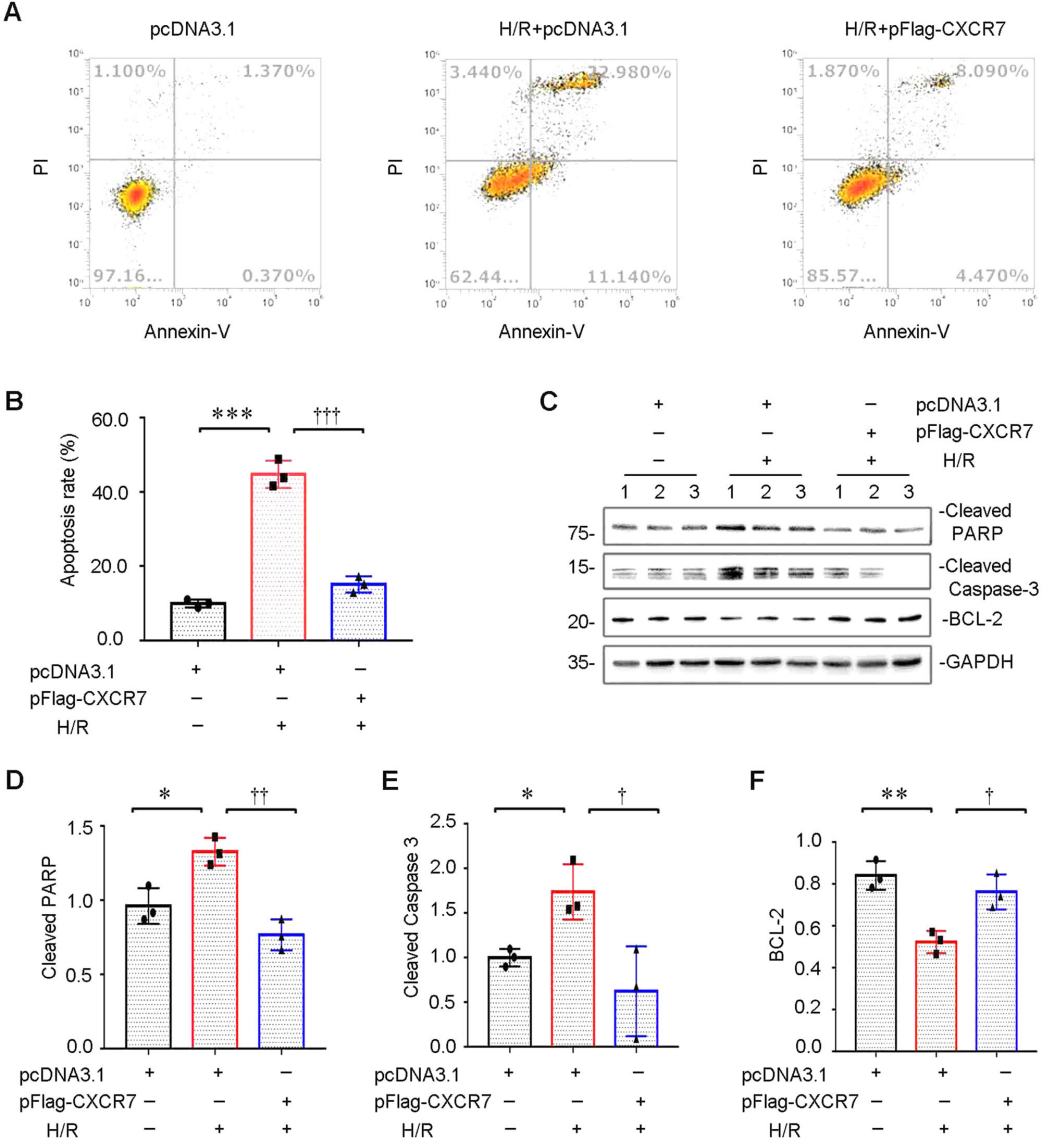
CXCR7 overexpression inhibits HR-induced HK-2 cell apoptosis. **A**, **B** Graphical representation of apoptosis. Apoptotic cells were detected by Annexin V/PI staining and analysed by flow cytometry (**A**). The percentage of apoptotic cells is shown in a bar chart (**B**). ***p < 0.001 versus the pcDNA3.1 group, †††p < 0.001 versus the pcDNA3.1 + H/R group, *n* = 3. **C**–**F** Western blots (**C**) and graphical representations of (**D**) cleaved PARP-1, (**E**) cleaved Caspase-3, and (**F**) BCL-2 protein expression levels in different groups are presented. *P < 0.05, **P < 0.01, †p < 0.05, ††p < 0.01, *n* = 3

Flow cytometry data showed that CXCR7 overexpression significantly reduced the H/R-induced apoptosis rate of HK-2 cells (Fig. 2A, B). Western blot data showed that CXCR7 overexpression inhibited the expression of the apoptosis-related molecules cleaved PARP and cleaved caspase 3 and the protein level of BCL-2 (Fig. 2C–F).

### CXCR7 Overexpression Promotes Autophagy

**Fig. 3:**
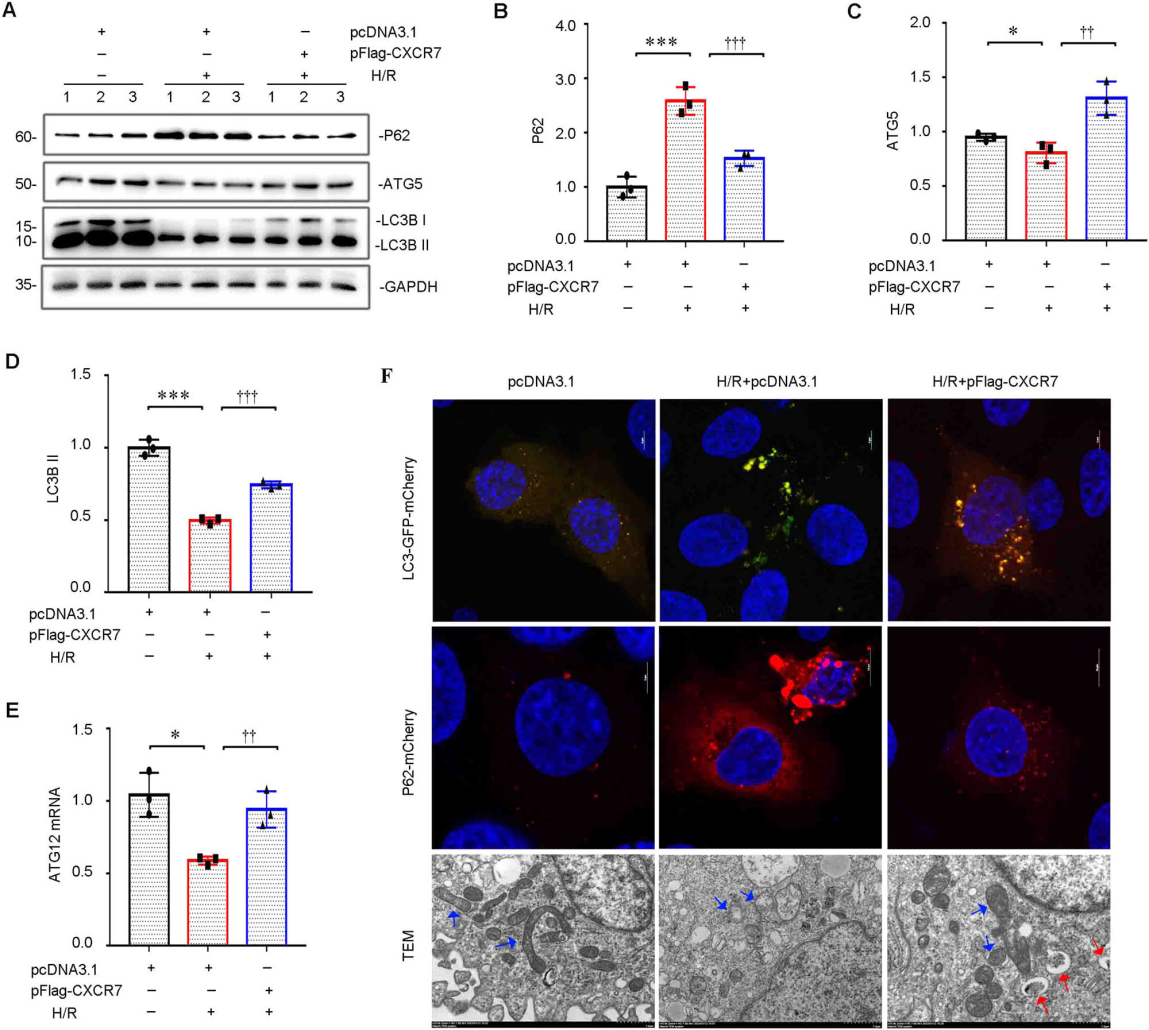
CXCR7 promotes HK-2 cell autophagy. **D** Western blots (**A**) and graphical representations of (**B**) P62, (**C**) ATG5, and (**D**) LC3B II protein expression levels in different groups are presented. *P < 0.05, ***P < 0.001, ††p < 0.01, †††p < 0.001, *n* = 3. **E** Graphical representations showing the relative abundance of *ATG2* mRNA in the three groups. *P < 0.05, ††p < 0.01, *n* = 3. **F** Representative immunofluorescence staining (LC3 and P62) and TEM images for the three groups. Arrows indicate mitochondria (blue) and autophagy vesicles (red)

Western blot results showed that CXCR7 overexpression significantly upregulated the protein expression of autophagy-related ATG5 and LC3B II and inhibited the protein expression of P62, indicating that CXCR7 promoted autophagy (Fig. 3A–D). The qRT‒PCR results showed that CXCR7 overexpression significantly upregulated the mRNA expression of the autophagy-related gene *ATG2* (Fig. 3E). The autophagy flux results for LC3 indicated that CXCR7 significantly promoted autophagy. The immunofluorescence results showed that CXCR7 significantly inhibited the expression of P62. The TEM results showed that upregulating CXCR7 expression significantly promoted the formation of autophagic vacuoles (Fig. 3G). The above results indicated that CXCR7 significantly promoted autophagy.

### CXCR7 Inhibits Cell Apoptosis by Promoting Autophagy

**Fig. 4:**
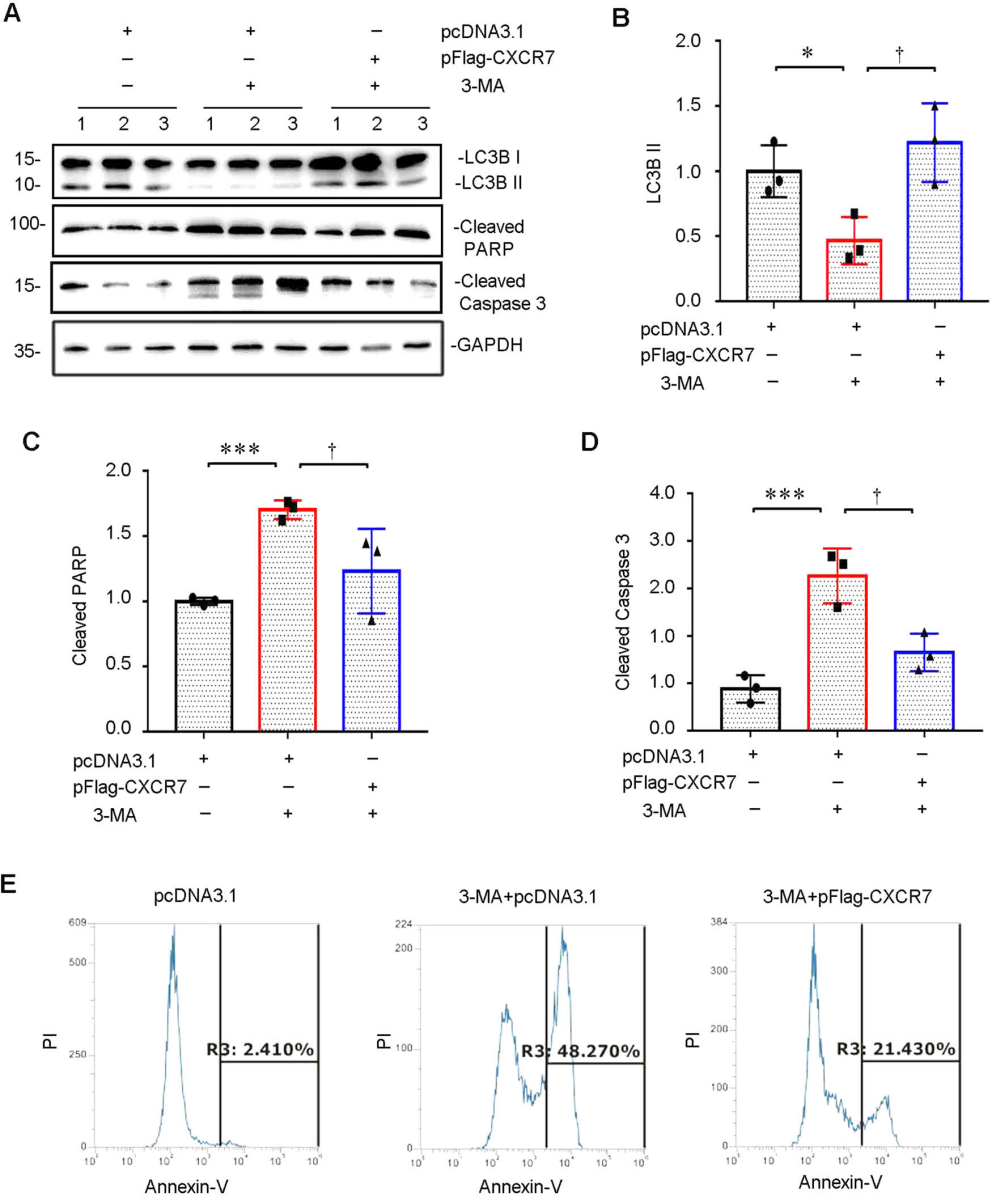
CXCR7 inhibits cell apoptosis by activating autophagy. (**A**–**D**) Representative graphs of protein expression levels of (**B**) LC3B II, (**C**) cleaved PARP-1, and (**D**) cleaved Caspase-3 are shown. *P < 0.05, ***P < 0.001, †p < 0.05, *n* = 3. **E** A representative parameter histogram of cell apoptosis is shown

Western blot and flow cytometry results showed that CXCR7 overexpression significantly promoted 3-MA-induced autophagy-induced damage and inhibited 3-MA-induced apoptosis (Fig. 4A–E). The above results indicated that CXCR7 might inhibit tubular epithelial cell apoptosis by promoting autophagy.

## Discussion

The development of AKI may be associated with a complex cascade of physio-pathological mechanisms and may not be determined by one specific process [1]. IRI is the most common cause of in-hospital AKI and is associated with increased morbidity and mortality [26]. The main pathological process involved in AKI is tubular injury. Recent studies have shown that multiple CXCR family members are closely related to autophagy [27–29]. However, the regulatory effect of CXCR7 on autophagy has not yet been reported. Herein, we revealed a counterintuitive finding that CXCR7 was downregulated in HK-2 cells under HR conditions (Fig. 1 A-D) and demonstrated that CXCR7 overexpression attenuated hypoxia-triggered tubular injury (Fig. 1E, F). Thus, CXCR7 is a possible candidate for alleviating the effects of kidney injury.

Previous studies have shown that CXCR7 activation plays an important protective role in ischaemic cells in hypoxic endothelial cells and AMI model mice by reducing apoptosis. Studies have also shown that in an AKI animal model, the upregulation of autophagy can alleviate TEC apoptosis [30]. In our study, we found that CXCR7 prevented hypoxia-triggered TEC damage by suppressing apoptosis (Fig. 2). Therefore, we hypothesized that upregulating autophagy by activating CXCR7 could effectively suppress TEC apoptosis.

A study suggested that basal autophagy is important for normal kidney homeostasis and that renal dysfunction and damage are more common in mouse kidneys that are deficient in autophagy-related gene expression. In addition, continuous autophagy activation exacerbates renal damage and triggers renal cell death pathways [31, 32]. We further confirmed that CXCR7 plays an important protective role against H/R-triggered TEC damage by promoting autophagy (Fig. 3). Pretreatment with 3-MA, an autophagy inhibitor, exacerbates tubular epithelial cell apoptosis and kidney damage [33]. Our current findings suggest that CXCR7 mediates autophagy by inhibiting cell apoptosis (Fig. 4). These findings indicate that CXCR7 may inhibit renal tubular epithelial cell apoptosis and damage by activating autophagy. Notably, our results, for the first time, confirm that targeting CXCR7 may be useful for treating AKI. However, future studies on a CXCR7-specific knock-in mouse model and the specific underlying molecular mechanism are needed.

## Conclusions

Taken together, our results showed that upregulating the expression of CXCR7 protected against renal tubular epithelial cell damage and apoptosis under H/R conditions, effects that were related to the activation of autophagy. Further understanding of the CXCR7-dependent autophagic process may provide a novel therapeutic approach for the treatment of AKI.

## Data Availability

No datasets were generated or analysed during the current study.
